# Organoid: a powerful tool to study lung regeneration and disease

**DOI:** 10.1186/s13619-021-00082-8

**Published:** 2021-04-26

**Authors:** Tiantian Lu, Yiyuan Cao, Peng Zhao, Shengxi Shen, Ying Xi

**Affiliations:** School of Life Science and Technology, ShanghaiTech University, Shanghai, China

## Abstract

Organoids are three-dimensional self-organizing structures formed by adult tissue stem cells or pluripotent stem cells. They recapitulate cell-cell, cell-niche interactions in tissue development, homeostasis, regeneration and disease, and provide an in vitro model for drug screening. This review summarizes the recent advances of organoid cultures derived from adult lung stem cells and human pluripotent stem cells, especially focusing on the organoids of the distal airway stem/progenitor cells. We also discuss the applications of organoids in studying lung regeneration and pulmonary diseases, including pulmonary fibrosis, airway diseases and Coronavirus disease 2019 (COVID-19).

## Background

The mammalian respiratory system is a tree-like structure consisting of trachea and branched airway tubes, and terminating in millions of air sacs called alveoli, where the gas exchange with the vasculature happens. Though the lung is a highly quiescent tissue with low steady-state cell turnover, it responds robustly after injury. As constantly exposed to airborne stimuli, such as cigarette smoke, pollutants, virus, and etc., the lung has evolved multifaceted tools of repair. It’s now known that depending on the type and severity of injury, regional stem/progenitor cells are activated (Hogan et al., 2014; Basil et al., 2020). Among those are airway basal cells which give rise to all the airway epithelial cells (Rock et al., 2009), club cells which can differentiate to ciliated cells (Rawlins et al., 2009), pulmonary neuroendocrine cells that give rise to club and ciliated cells (Song et al., 2012) and alveolar type II cells (AEC2s) as the stem cells in alveoli (Barkauskas et al., 2013). Recently, more evidence show that distal airway stem/progenitor cells, including bronchioalveolar stem cells (BASCs) co-expressing AEC2 and club cells markers (Kim et al., 2005; Liu et al., 2019), rare p63^pos^Krt5^neg^ cells (Vaughan et al., 2015; Yang et al., 2018; Xi et al., 2017), and H2-K1^high^ cells hiding among club cells (Kathiriya et al., 2020a), contribute to both airway and alveolar repair, all of which expended our knowledge of lung epithelial stem cells.

Stem-cell derived 3-dimentional self-organizing structures, named organoids are emerging as a powerful tool to study stem cells ex vivo. They recapitulate cell-cell and cell-niche interactions in development, homeostasis and disease, and can be scaled up for high throughput screening of small molecules that determine the cell fate. Besides, organoids derived from human cells exhibit great advantages in studying human epithelial stem cell biology and mimicking human diseases. Since the pandemic of COVID-19, human lung organoids have been quickly employed to study the pathobiology of SARS-CoV-2 infection in human lung epithelium and drug screenings against the virus infection were performed (Salahudeen et al., 2020; Han et al., 2020; Huang et al., 2020; Hou et al., 2020). Therefore, lung organoids have become an indispensable tool for in vitro modeling of organ development, regeneration and disease.

Since the first organoid culture from airway basal cells (Rock et al., 2009), lung organoids have successfully grown from adult stem cells, human pluripotent stem cells (hPSCs) including embryonic stem cells (ESCs) and induced pluripotent stem cells (iPSCs). Previous reviews have summarized very nicely the different culture systems using airway basal cells, secretory cells, AEC2s, BASCs, and hPSCs in detail (Barkauskas et al., 2017; Nikolic & Rawlins, 2017; Nadkarni et al., 2016; van der Vaart & Clevers, 2020; Tian et al., 2020), which we are not going to reiterate. In this review, we discuss the recent advances of lung organoid systems, focusing on the findings from organoids, especially that from distal airway stem/progenitor cells. We further review the applications of organoid technologies in studying lung regeneration and diseases, including pulmonary fibrosis, airway diseases, cancer and infectious diseases. Given human lung organoids faithfully mimic virus infection in living organisms, we also summarize the current studies of SARS-CoV-2 infection using human lung organoids.

## Organoids from airway basal cells

Most of human lung airways is lined by pseudostratified epithelium consisting of airway basal cells, secretory, ciliated, tuft and neuroendocrine cells, whereas in mice, the pseudostratified epithelium is confined to the trachea and main bronchi (Hogan et al., 2014). Thus, basal cells are present throughout the airways in human lungs, including the small bronchioles of 1 mm in diameter, but restricted in trachea and main bronchi in mouse. Basal cells make up around 30% of the pseudostratified lung epithelium and adhere closely to the basal lamina (Boers et al., 1998). They have self-renewal capacity and can give rise to secretory and ciliated luminal cells during homeostasis and repair (Rock et al., 2009). The characteristic genes expressed in basal cells include p63, cytokeratin 5 (Krt5), nerve growth factor receptor (NGFR) and Integrin α6.

**Fig. 1:**
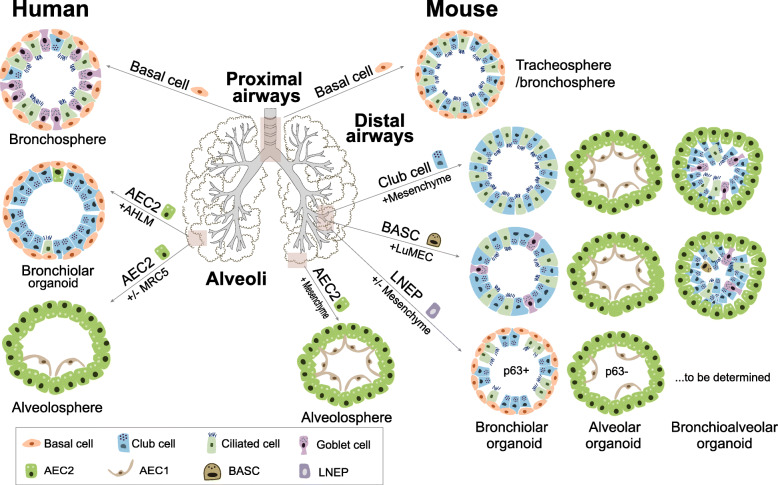
Schematic of adult human and mouse lung stem cell-derived organoids**.** Human and mouse airway basal cells form tracheosphere or bronchosphere when cultured in 3D Matrigel (Rock et al., 2009; Danahay et al., 2015). Human and mouse AEC2s form alveolosphere with or without the support of fetal lung fibroblasts MRC5 (Barkauskas et al., 2013; Youk et al., 2020), whereas human AEC2s give rise to bronchiolar organoids in co-culture with adult human lung mesenchymal cells (AHLM) (Kathiriya et al., 2020b). In mouse distal airways, club cells and BASCs generate three distinct colony types when co-cultured with mesenchymal cells or mouse lung endothelial cells (LuMECs) (Lee et al., 2014; Lee et al., 2017). p63^pos^ LNEPs form bronchiolar organoids, while p63^neg^ LNEPs mainly form alveolar organoids (Xi et al., 2017; Kathiriya et al., 2020a; Cassandras et al., 2020). It remains to be determined whether there is stem/progenitor cell population in the distal human airways, as in mice

The 3D organoids derived from trachea basal cells are called tracheosphere, while that from bronchi or large airways are named bronchosphere (Barkauskas et al., 2017). The first tracheosphere was from Krt5-GFP^pos^ mouse tracheal basal cells, forming spheres with a visible lumen within 1 week in Matrigel (Rock et al., 2009). By 14 days, a pseudostratified epithelium formed with an outer layer of p63^pos^ Krt5/Krt14^pos^ basal cells and an inner layer of Krt8^pos^ luminal secretory and ciliated cells (Fig. 1). The basal cells can be isolated by flow sorting using NGFR, Integrin α6 and cultured in transwell inserts or multiwell plates with or without the support of stromal cells (Danahay et al., 2015; Rock et al., 2011; Tata et al., 2013; Hild & Jaffe, 2016). Human bronchospheres from p63^pos^ NGFR^pos^ Integrin α6^pos^ airway basal cells are mainly composed of basal cells and two major differentiated cells, goblet cells and ciliated cells, recapitulating the cellular complexity of human conducting airways (Danahay et al., 2015; Hild & Jaffe, 2016).

Tracheospheres and bronchospheres have been used for screening secreted factors that influence basal cell self-renewal and differentiation. A number of factors that modulate basal cell fate were identified using organoid cultures, including interleukin 6 (IL6) which promotes differentiation of ciliated cells versus club cells through signal transducer and activator of transcription 3 (STAT3) signaling (Tadokoro et al., 2014), interleukin 13 (IL-13) and interleukin 17A (IL-17A) which induce goblet cell production at the expense of ciliated cells (Danahay et al., 2015),mimicking goblet cell metaplasia (GCM) phenotype. GCM represents a common key feature of many airway diseases, including asthma, chronic obstructive pulmonary disease (COPD) and cystic fibrosis. Overproduction of mucus and the inability to clear increased mucus can lead to airflow obstruction, mucostasis and ultimately death (Kuyper et al., 2003; Hogg et al., 2004). Notch isoform specific blocking antibody screen in bronchospheres leads to the finding that Notch2 is required for cytokine-induced goblet cell metaplasia and Notch2 inhibition promotes basal cell differentiation toward a ciliated cell fate (Danahay et al., 2015; Pardo-Saganta et al., 2015). Therefore, Notch2 was proposed as a new therapeutic target for goblet cell metaplasia in airway diseases, consistent with Jagged blockade reversing goblet cell metaplasia (Lafkas et al., 2015). Inflammatory cytokines promote basal cell differentiation toward goblet cell in human bronchospheres, which mimics GCM and proves to be useful for screening secreted proteins, small molecules and drugs in search of airway disease therapeutics.

## Organoids from alveolar type II cells

The alveolar epithelium is composed of two types of epithelial cells, AEC2s and alveolar epithelial type I cells (AEC1s). AEC1s are large squamous cells that cover about 95% of the alveoli surface area and account for gas exchange function, whereas AEC2s are cuboidal cells and function as stem cells in alveoli, repopulating both AEC2s and AEC1s after injury. Primary AEC2s enriched by surface markers (EPCAM^pos^ HTII-280^pos^ for human, EPCAM^pos^ Sca1^neg^ or EPCAM^med^ for mouse) or lineage-tracing approaches (Sftpc-CreERT2; Rosa-tdTomato) are co-cultured with support cells such as PDGFRα^pos^ lung fibroblasts (Barkauskas et al., 2013), Mlg cell lines (Chen et al., 2012), EPCAM^neg^ Sca1^pos^ lung mesenchymal cells (McQualter et al., 2010) or lung endothelial cells (Lee et al., 2014) in Matrigel. 3D spheroids develop within about 14 days, with AEC2s on the outside and AEC1s inside the lumen (Fig. 1). The derived organoids are called alveolosphere (Barkauskas et al., 2013). To date, fibroblast-free cultures are established using fibroblast-expressed ligands and small molecule inhibitors (Youk et al., 2020; Shiraishi et al., 2019). Human AEC2s can be maintained in a feeder-free, long-term 3D culture system using chemically defined medium, containing the factors that have been implicated in lung development (Youk et al., 2020), CHIR99021, R-spondin1, fibroblast growth factor 7 (FGF7), fibroblast growth factor 10 (FGF10), epidermal growth factor (EGF), Noggin and SB431532.

Alveolosphere has been widely used to study the regulation of AEC2 proliferation, AEC2 to AEC1 differentiation (Sun et al., 2019), AEC2-fibroblast crosstalk (Lee et al., 2017; Nabhan et al., 2018; Wang et al., 2018; Zepp et al., 2017), and AEC2-macrophage crosstalk (Lechner et al., 2017; Choi et al., 2020). It works successfully for screening small-molecule inhibitors (Sun et al., 2019; Katsura et al., 2019). Recently, scRNAseq analysis of alveolosphere uncover a previously uncharacterized transient state during AEC2 to AEC1 differentiation (Choi et al., 2020; Kobayashi et al., 2020). This transitional state also exists during mouse alveolar regeneration after bleomycin injury and correlates with the abnormal basal-like cells in human lungs with idiopathic pulmonary fibrosis (IPF) (Choi et al., 2020; Kobayashi et al., 2020; Strunz et al., 2020). Though the transitional cells identified by three independent studies were termed pre-alveolar type-1 transitional cell state (PATS) (Kobayashi et al., 2020), damage-associated transient progenitors (DATPs) (Choi et al., 2020), or Krt8^+^ alveolar differentiation intermediate (ADI) (Strunz et al., 2020), they share similar sets of signature genes, including Krt8 and Claudin 4 (Cldn4), low levels of AEC2 and AEC1 markers, and feature TP53, Transforming growth factor β (TGFβ), nuclear factor κB (NF-κB) activation, DNA damage response and senescence, phenocopying the aberrant basaloid cells identified in IPF lungs (Adams et al., 2020; Habermann et al., 2020). These cells also appear similar to the intermediate cells derived from AEC2s of CDC42 null mice after pneumonectomy which show a progressive lung fibrosis (Wu et al., 2020), suggesting persistence of transitional cells may mediate lung fibrosis. Further study of these transitional cells may largely advance our understanding of the pathogenesis of pulmonary fibrosis.

Though mouse alveolar organoids have been well characterized, the human alveolar organoids need further study. The first adult human alveolar organoid of EPCAM^pos^ HTII-280^pos^ human AEC2s and fetal human lung fibroblast cell line MRC5 show that hAEC2s are capable of clonal growth in vitro, but there is no differentiation into AEC1s^6^. In similar culture conditions, a subset of Wnt-responsive hAEC2s (HTII-280^pos^ TM4SF1^pos^), exhibit higher clonogenic potential than bulk hAEC2s and can differentiate into AEC1s^50^ (Fig. 1). A recent study using adult primary human lung mesenchymal cells (hHLMs) instead of fetal human lung fibroblasts demonstrate that hAEC2s can transdifferentiate into Krt5^pos^ basal cells, suggesting hAEC2s have different plasticity than their mouse counterparts and also highlighting that the nature of mesenchymal cells in the co-culture system determines the fate of epithelial stem/progenitors (Kathiriya et al., 2020b). Interestingly, hAEC2s/hHLMs organoids show a gradual emergence of alveolar-basal intermediate cells, co-expressing basal cell and AEC2 markers, which give a way to mature basal cells and subsequently club and ciliated cells. These Surfactant protein C (SPC)^low^ ABCA3^low^ KRT17^pos^ KRT5^neg^ intermediate cells exist in IPF lungs, consistent with the aberrant basaloid cells mentioned earlier (Adams et al., 2020; Habermann et al., 2020), implicating this organoid system mimics a gradual shift from hAEC2s to pathogenic IPF-like epithelium and thus may be a useful platform to study human epithelial metaplasia in IPF.

## Organoids from airway secretory cells

In mouse bronchioles, club cells expressing Scgb1a1 function as long-term progenitors. They can self-renew and give rise to ciliated cells during postnatal growth and adult homeostasis (Rawlins et al., 2009; Evans et al., 1976). A subpopulation of club cells, named variant club cells, are defined by the location near neuroendocrine bodies (NEBs) and low expression of cytochrome Cyp2f2, are resistant to naphthalene injury and thus can repopulate mouse bronchiolar epithelium following this injury (Reynolds et al., 2000; Hong et al., 2001). Recently, Upk3a was identified as a unique marker for variant club cells (Guha et al., 2012; Guha et al., 2017). Upk3a expressing cells generate both club and ciliated cells during development, adult airway maintenance and after naphthalene injury. These cells also contribute to alveolar repair after bleomycin injury to some degree (Guha et al., 2017). It is currently unclear whether human club cells function as stem/progenitor cells.

In organoid culture, Scgb1a1-Cre labeled cells show multi-lineage differentiation when co-cultured with Lgr6^pos^ or Lgr5^pos^ mesenchymal cells, displaying bronchiolar, alveolar, or bronchioalveolar colonies (Lee et al., 2017) (Fig. 1). Bronchiolar colonies are large and round, having a single lumen consisted of secretory and ciliated cells; alveolar colonies are small and dense with AEC2s in the outer layer and AEC1s in the inner layer; bronchioalveolar colonies are mixed with airway and alveolar epithelial cells. Consistent with their distinct location in the lung with majority of Lgr6^pos^ cells surrounding airway epithelia and Lgr5^pos^ cells largely located in alveolar regions, Lgr6^pos^ and Lgr5^pos^ cells promote airway or alveolar differentiation of Scgb1a1 expressing cells, respectively. Later, it was found that Scgb1a1^pos^ cell population in the bronchioles is a heterogenous population, including club cells, bronchio-alveolar stem cells (BASCs) and H2-K1^high^ club cell-like progenitors, which will be discussed later.

## Organoids from multipotent distal airway progenitors

Studies from several groups demonstrate the existence of distal airway stem/ progenitors exhibiting both alveolar and airway differentiation potential (Kim et al., 2005; Chen et al., 2012; McQualter et al., 2010; Chapman et al., 2011). BASCs located at the bronchioalveolar duct junction is the first identified adult lung stem cell population displaying the binary potential (Kim et al., 2005). BASCs co-express markers of club cells and AEC2s and maintain stable cell number during homeostasis (Liu et al., 2019). They give rise to club and ciliated cells after naphthalene mediated bronchiolar injury and contribute to AEC2s and AEC1s after bleomycin induced alveolar injury.

Single BASC develop multilineage lung organoids in co-culture with primary mouse lung endothelial cells (LuMECs), which support BASCs self-renewal and differentiation (Lee et al., 2014). Three colony types arise in BASC/LuMEC organoids in vitro and after subcutaneous injection in mice: bronchiolar colonies expressing ciliated cells, club and goblet cell markers, alveolar colonies expressing AEC2 and AEC1 markers, and bronchioalveolar colonies mixed with club cells, goblet cells, ciliated cells, AEC2s, and Scgb1a1/SPC dual positive cells (Fig. 1). This organoid co-culture provides a versatile flatform to elucidate the cross-talk between epithelial and endothelial cells and show that endothelial cells govern BASC differentiation to alveolar lineages through bone morphogenetic protein 4 (BMP4)- nuclear factor of activated T cell c1 (NFATc1)- thrombospondin-1 (TSP1) signaling axis. Epithelial derived BMP4 activates NFATc1 signaling in LuMECs through Bmpr1a to induce TSP1 expression, which in turn directs BASCs differentiation to alveolar cell fate at the expense of bronchiolar cell fate. Consistently, bleomycin induced alveolar injury triggers BMP4 induction in BASCs and AEC2s, subsequently upregulating TSP1 expression in lung endothelial cells to drive alveolar repair.

Later, an in vivo organoid assay was developed and demonstrated the multipotential of Integrin α6β4^pos^ SPC^neg^ adult lung epithelial cells (Chapman et al., 2011). These adult epithelial cells were mixed with E14.5 lung single-cell suspension, placed under renal capsules and developed lung organoids within 6 days, displaying either SPC^pos^ saccules or Scgb1a1^pos^ airway-like structures. These Integrin α6β4^pos^ SPC^neg^ epithelial cells expand and give rise to AEC2s after bleomycin injury, providing a direct evidence that maintenance of AEC2s after alveolar injury involve progenitor cell differentiation, besides expansion of the pre-existing AEC2s.

Subsequently, a distal airway stem/progenitor population contributing to mouse alveolar repair following severe injury was identified. Though different terms were used by different groups, lineage-negative epithelial progenitors (LNEPs) or distal airway stem cells (DASCs), these cells are rare p63^pos^ Krt5^neg^ cells residing in distal airways and expand and mobilize to generate Krt5^pos^ pods after H1N1 PR8 infection (Vaughan et al., 2015; Zuo et al., 2015; Kumar et al., 2011). Later it was found that LNEPs consist of both p63^pos^ Krt5^neg^ and p63^neg^ cells (Xi et al., 2017). The p63^pos^ cells appear to be holdovers from embryonic intrapulmonary p63^pos^ progenitors and give rise to Krt5^pos^ basal-like cells (Yang et al., 2018). Though initially protective by quickly restoring the epithelial barriers, these Krt5^pos^ basal-like cells have limited potential to differentiate into AEC2s, thus insufficient to restore lung function, leading to permanent cystic structures in alveoli (Vaughan et al., 2015; Yang et al., 2018; Xi et al., 2017; Zuo et al., 2015; Zacharias et al., 2018; Kanegai et al., 2016). On the other hand, the p63^neg^ progenitors including the H2-K1^high^ club cell-like progenitors can give rise to AEC2s and AEC1s, contributing to alveolar regeneration in the case of severe lung injury (Xi et al., 2017; Kathiriya & Brumwell, 2020). Though the heterogeneity has not been fully resolved and distinct markers are lacking, LNEPs are hiding among the cells expressing Integrin α6β4 and Sox2 in distal airways. Lineage tracing using Sox2-Cre and orthotopic transplantation of the enriched Integrin β4^pos^ population clearly demonstrated the binary potential of LNEPs to reconstitute damaged alveolar barriers with either alveolar (SPC^pos^) or metaplastic (Krt5^pos^) epithelium (Vaughan et al., 2015).

The local environment and niche factors determine the distal airway progenitor cell fate and the outcome of alveolar regeneration. Local lung hypoxia induced by extensive epithelial death activates Notch signaling to promote metaplastic differentiation of LNEPs to Krt5^pos^ basal-like cells through hypoxia inducible factor 1 (HIF1α); whereas Wnt/β-catenin pathway antagonizes hypoxia and Notch signaling to promote alveolar regeneration (Xi et al., 2017). HIF1α deletion or enhanced Wnt/β-catenin activity block Krt5 activation and promote alveolar gas exchange. Signals from mesenchyme function as specialized niche directing the response of adjacent airway epithelium. After fibrotic injury, activated Gli1^pos^ mesenchymal stromal cells form a pathological niche to promote the metaplastic Krt5 differentiation of Sox2^pos^ airway progenitors by upregulating BMP antagonists (Cassandras et al., 2020). Restoring BMP4 attenuated the metaplastic airway progenitor differentiation concurrent with an increase of alveolar cell fate and lung function recovery, consistent with earlier study showing that BMP4 promotes alveolar repair (Lee et al., 2014).

Organoids derived from Sox2^pos^ or Integrin β4^pos^ distal airway cells display alveolar or bronchiolar differentiation with or without the supporting cells (Xi et al., 2017; Cassandras et al., 2020; Kathiriya & Brumwell, 2020) (Fig. 1). Given Sox2 and integrin β4 label a heterogenous population of all the airway epithelial cells, including but not limited to BASCs, LNEPs and H2-K1^high^ club cell-like progenitors, further characterization of the distal airway progenitors is needed. It remains to be determined whether BASCs, LNEPs and H2-K1^high^ progenitors represent overlapping or similar populations of cells and how these populations contribute to alveolar regeneration after injury or in chronic diseases. Moreover, it is unknown whether distal airway progenitor population that mobilize after injury and contribute to alveolar repair exist in human lungs. We expect single-cell RNAseq analysis combined with human lung organoids to provide more insights. Of note, we and others have noticed the cell differentiation fate change during the time course of culture and transitional states exist, underscoring the importance of time-course study at single cell level in organoids.

## Organoids from human pluripotent stem cells (hPSCs)

Interspecies differences highlight the necessity of human lung studies, which unfortunately is hindered by availability and limited access to human tissues. In the past few years, series of protocols have been developed to differentiate hPSCs into pulmonary fate successfully. The general process involves stepwise differentiation from hPSCs to definitive endoderm, anterior foregut endoderm and ventralized anterior foregut endoderm, which results in a mixed population of ventral anterior foregut endoderm-like cells, including lung lineage cells expressing NKX2.1 and Sox2 (Dye et al., 2015; Jacob et al., 2017; Yamamoto et al., 2017; Gotoh et al., 2014; Konishi et al., 2016; Huang et al., 2014). These progenitors could be further differentiated towards airway or alveolar fate by modulating signaling pathways. Temporal modulation of Wnt activity promote maturation of AEC2s^64^. More recently, multigerm layer 3D lung organoids have been generated, in which lung epithelium and mesenchyme coexist (Dye et al., 2015; Chen et al., 2017). Studies have shown that hPSC-derived lung organoids generate more mature airway epithelium after transplanted into mice.

hPSCs have been adopted for the study of human lung physiology and pathology in vitro. As they retain the potential to differentiate into every cell type of the body, hPSC-derived lungs cells provide an accessible and a potentially unlimited cell source for understanding human organogenesis, diseases modelling and drug screening. Chen et al. generated lung bud organoids (LBOs) from hPSCs that contain mesoderm and pulmonary endoderm and develop into branching airway and early alveolar structures in 3D Matrigel and after kidney capsule xenotranplantation (Chen et al., 2017). The LBO organoids recapitulate the development of late second trimester of human gestation and reproduce features of respiratory syncytial virus (RSV) infection in human lungs. It also shows evidence of fibrosis associated with Hermansky-Pudlak Syndrome (HPS) upon CRISPR/Cas9-mediated deletion of HPS1, HPS2, or HPS4 gene (Chen et al., 2017; Strikoudis et al., 2019). HPS is an autosomal recessive genetic disease caused by abnormal biogenesis and trafficking of lysosome-like organelles (Vicary et al., 2016). Some patients with HPS develop lung fibrosis, sharing similar histological patterns with IPF. HPS deficiency in organoids leads to accumulation of mesenchymal cells and increased expression of matrix proteins including collagen I, collagen III and fibronectin, mimicking IPF.

Patient specific iPSCs allow in vitro modeling of human genetic diseases and drug screening. Infants with homozygous SFTPB^121ins2^ mutation develop neonatal respiratory distress. The dermal fibroblasts from patients were reprogrammed to iPSC line and then differentiated to AEC2s, showing decreased SFTPB expression and deficient processing of surfactant proteins, which can be restored by CRISPR/Cas9-mediated correction of SFTPB^64^. Cystic fibrosis (CF) is a rare genetic disease, caused by mutations in the cystic fibrosis transmembrane conductance regulator (CFTR) gene which functions as an anion channel across the cell membrane. cAMP inducing agent forskolin induces transportation of anions and fluids to organoid lumen and thus a CFTR-dependent swelling of organoids (Dekkers et al., 2013). CF patient-specific iPSC-derived airway organoids show inability to swell upon forskolin treatment, and this is rescued by correction of the mutation (McCauley et al., 2017). Therefore, iPSC organoids provide a robust functional assay for CFTR gene and facilitate personalized medicine development for cystic fibrosis.

## Human lung organoids for disease modeling

As mentioned above, organoid cultures from hPSCs or adult stem cells can model certain aspects of human lung diseases, including COPD, asthma, cystic fibrosis, IPF and virus infections (Danahay et al., 2015; Kobayashi et al., 2020; Strunz et al., 2020; Jacob et al., 2017; Chen et al., 2017; McCauley et al., 2017). Human lung organoids can also be generated from mixed populations of primary cells in normal or diseased lungs. Human airway organoids derived from broncho-alveolar biopsy or bronchoalveolar lavage are capable of long-term expansion (> 1 year) and contain all the major epithelial cell types of pseudostratified airways, including basal cells, ciliated cells, club and goblet cells (Sachs et al., 2019). These airway organoids allow modeling of cystic fibrosis, lung cancer and infectious pulmonary diseases, such RSV or SARS-CoV-2 infection, the latter of which will be reviewed in a separate section.

Infection of airway organoids with RSV showed specific virus-host interactions, epithelial remodeling and recruitment of neutrophils in co-culture, recapitulating the disease features (Sachs et al., 2019). Airway organoids derived from cystic fibrosis patients present a thicker layer of mucus and reduced swelling following forskolin treatment compared to wild-type airway organoids, recapitulating aspects of cystic fibrosis phenotypes. The patient-derived organoids respond to CFTR modulators, allowing personalized drug screenings.

Lung cancer organoids have been established recently from needle biopsies or resections of human lung tumors and can be maintained in long-term cultures (> 3 months) (Sachs et al., 2019; Shi et al., 2020; Kim et al., 2019; Li et al., 2020; Neal et al., 2018). Unlike cancer cell lines that are largely homogenous and lack cell-cell interactions in 3D environment, or patient-derived xenograft (PDX) models that are time-consuming, costly and have a low success rate though retaining tumor heterogeneity and microenvironment, patient-derived cancer organoids recapitulate the histopathological and genetic characteristics of the original tumors and the success rate is higher than that of PDX (Huo et al., 2020; Wang et al., 2020). Thus, lung cancer organoids provide a valuable tool for biomarker identification, high-throughput drug screening and prediction of patient-specific drug response in clinic.

## Human lung organoids for SARS-CoV-2 study

Human lung organoid serves as a valuable preclinical model to study COVID-19 pathobiology and therapeutic development. So far, several groups have developed the organoid cultures to study the infection response to SARS-CoV-2. Youk et al. established a 3D model to grow FACS sorted human AEC2s and airway epithelial cells to organoids and then broke them into pieces to enhance the access of virus to the apical cell surfaces (Youk et al., 2020). They found that SARS-CoV-2 productively infects AEC2s, resulting in an innate immune response. Mulay et al. employed alveolospheres of FACS sorted distal lung epithelial cells mixed with MRC5 cells and revealed that infection triggers both cell-autonomous and non-cell-autonomous apoptosis that may contribute to alveolar injury (Mulay et al., 2020). They also examined infection in air-liquid interface (ALI) cultures from proximal airway epithelial cells and found the virus targeted mainly ciliated cells in airway epithelium, consistent with the findings in COVID-19 autopsy lungs (Hou et al., 2020).

Salahudeen et al. developed a chemically defined culture of human peripheral lung tissue into both cystic AEC2 organoids and solid basal organoids, which have lumens lined by club and ciliated cells (Salahudeen et al., 2020). An apical-out suspension culture polarization method was used to facilitate SARS-CoV-2 access to luminal cells. They identified club cells as a novel target for SARS-CoV-2. Tindle et al. were able to develop lung organoids with all the 6 major epithelial cell types from distal human lung, including AEC2s, AEC1s, basal cells, club cells, goblet cells and ciliated cells (Tindle et al., 2020). The organoids were then dissociated and seeded as 2D monolayer in the apical chamber of transwells for virus infection. They demonstrated that the mixed cellularity of proximal and distal lung is important for viral and host immune response.

hPSCs derived lung organoids are also permissive to SARS-CoV-2 infection both in culture and in vivo, and mimic the inflammatory response in human COVID-19 (Han et al., 2020). Han et al. conducted high-throughput screen of FDA-approved drugs and identified several drugs that inhibit SARS-CoV-2 entry. Duan et al. developed a co-culture system of lung cells and macrophages from the same hPSC line allowing to study the pro- and anti-inflammatory macrophages in the host-pathogen interaction and immune response (Duan, 2020). Huang et al. generated an ALI culture of human iPSC-derived AT2-like cells (iAT2s) for apical infection and revealed an epithelial-intrinsic NF-κB-mediated innate immune response after SARS-CoV-2 infection (Huang et al., 2020).

Given most of the SARS-CoV-2 infection studies in organoids do not include immune cells, we anticipate that introducing immune cells into the cultures will largely advance our understanding of the pathogenesis of SARS-CoV-2 infection. The access of virus should be considered while evaluating which cell population can be infected, as the luminal epithelial cells in the 3D organoids are buried inside which is not the case in vivo.

## Conclusions and future directions

Lung organoids have proven to be a versatile and powerful tool to model development, homeostasis, regeneration and diseases. The organoids leverage the self-renewal and differentiation capability of stem cells to form organized structures, but the behavior of stem cells is also controlled by the microenvironment, including the cells in co-culture, extracellular matrix (ECM) substrates, molecules added to the system, and etc. However, the current versions of lung organoids are mainly comprised of epithelial cells. The absence of non-epithelial cells fails to recapitulate the architecture of real organ. Incorporation of mesenchymal cells in organoids has already been shown to promote self-renewal and direct the stem cell differentiation (Barkauskas et al., 2013; Kathiriya et al., 2020b; Cassandras et al., 2020). Macrophages are also reported to support AEC2s in 3D cultures (Lechner et al., 2017). Therefore, introducing non-epithelial cells such as immune cells, endothelial cells and mesenchymal cells into the cultures will better recapitulate the complexity of in vivo structure and function and advance our understanding of the interplay between stem cells and the dynamic environment. Furthermore, the transplantation of organoids in mice could potentially provide a better in vivo environment to evaluate stem cell behavior and function.

Besides the cells in co-culture, the components in growth medium and ECM play key roles in regulating stem cell behavior. The culture media often contain fetal bovine serum (FBS) or bovine pituitary extract (BPE), whereas matrix usually rely on Matrigel or basement membrane extracts (BME), all of which consist of complex growth factors and have significant lot-to-lot variability. Recently, chemically defined medium with growth factors and small molecule inhibitors has been established for organoid cultures (Salahudeen et al., 2020; Youk et al., 2020; Sachs et al., 2019). The components of ECM and the mechanical forces need rigorously examined in future studies.

Despite the fast-growing knowledges of mouse distal lung stem/progenitor cells, the parallel population in human remains unclear. Studies have demonstrated significant cellular and molecular differences between mouse and human lungs, highlighting the importance of studying human-specific mechanisms that are essential for understanding human diseases. Therefore, establishing human-derived organoids that recapitulate the complexities of human lung biology is crucial.

Patient-derived organoids, such as cancer organoids or CF organoids have proven to faithfully mimic disease. However, currently, normal cells are used to establish organoids to model chronic pulmonary diseases such as fibrosis and COPD, which may miss important pathological characteristics of these diseases. Future studies using cells freshly isolated from late-stage diseased lungs will largely enhance our understanding of human lung diseases and make it possible to carry out drug development and monitor patient-specific drug response. Besides, applying genome editing technologies such as CRISPR/Cas9 opens up the possibility to model genetic diseases.
